# Impact of the Type of Continuous Insulin Administration on Metabolism in a Diabetic Rat Model

**DOI:** 10.1155/2016/8310516

**Published:** 2016-07-18

**Authors:** A. Schaschkow, C. Mura, S. Dal, A. Langlois, E. Seyfritz, C. Sookhareea, W. Bietiger, C. Peronet, N. Jeandidier, M. Pinget, S. Sigrist, E. Maillard

**Affiliations:** ^1^UMR DIATHEC, EA 7294, Centre Européen d'Etude du Diabète, Université de Strasbourg (UdS), Boulevard René Leriche, 67200 Strasbourg, France; ^2^Department of Endocrinology, Diabetes, and Metabolic Diseases, Pôle NUDE, Hôpitaux Universitaires de Strasbourg (HUS), 67000 Strasbourg Cedex, France

## Abstract

Exogenous insulin is the only treatment available for type 1 diabetic patients and is mostly administered by subcutaneous (SC) injection in a basal and bolus scheme using insulin pens (injection) or pumps (preimplanted SC catheter). Some divergence exists between these two modes of administration, since pumps provide better glycaemic control compared to injections in humans. The aim of this study was to compare the impacts of two modes of insulin administration (single injections of long-acting insulin or pump delivery of rapid-acting insulin) at the same dosage (4 IU/200 g/day) on rat metabolism and tissues. The rat weight and blood glucose levels were measured periodically after treatment. Immunostaining for signs of oxidative stress and for macrophages was performed on the liver and omental tissues. The continuous insulin delivery by pumps restored normoglycaemia, which induced the reduction of both reactive oxygen species and macrophage infiltration into the liver and omentum. Injections controlled the glucose levels for only a short period of time and therefore tissue stress and inflammation were elevated. In conclusion, the insulin administration mode has a crucial impact on rat metabolic parameters, which has to be taken into account when studies are designed.

## 1. Introduction

Glycaemia regulation is closely controlled by beta cells (*β*-cells), which routinely monitor blood glucose increases and control insulin release. Blood insulin rise triggers glucose absorption and storage in hepatic, adipose, and muscle cells. The rapid regulation by *β*-cells permits the blood glucose to return to a normal fasting level within an hour after eating [1]. Uncontrolled glycaemia and a high level of blood glucose are associated with diabetes, in particular type 1 diabetes, which is an autoimmune disease triggering the specific destruction of *β* cells. The lack of insulin cannot be compensated by other hormones since insulin is the unique hypoglycaemic hormone. Moreover, because of its sensitivity to the digestive tracts [2], insulin needs to be injected by the parenteral route to be biofunctional.

The main issue with insulin injection self-management is that the proper tight regulation of glycaemia to that of *β*-cells is dependent upon the compliance of the patient. The right dose of insulin needs to be administrated at the right time and it is based mainly on experience and training. In order to mimic *β*-cell action, different types of insulin are available: rapid-, long-, and medium-acting insulin types. Type 1 diabetic patients typically require some basal level of insulin, provided by a long-acting insulin (given once or twice a day), as well as short- and rapid-acting insulin types to cover meals (bolus insulin). The compliance of the patient can be improved by the use of a pump to allow continuous insulin release. In fact, pumps can release a small amount of rapid insulin continuously as a basal level, and a bolus can be ordered directly via the pump at meal time. Administration of a continuous release of rapid-acting insulin is more reliable than multiple injections of the long-acting insulin. Indeed, in numerous clinical studies [3–8], continuous release has shown improvements of blood glucose values [9] and HbA1c levels [10], as well as decreases in global inflammation [11, 12] and oxidative stress [13] levels in plasma of diabetic patients equipped with pumps.

The different impact of the two modes of insulin administration is well documented in humans but barely so in rodent models, which is the main model used in research. Since the differences between injection and continuous delivery in humans are so important in terms of global inflammation and oxidative stress, the differences are likely to be equally as important in rodent models, and the choice of insulin administration could interfere with the results of a study, especially with rats which do not have a specific meal time and eat all night long. Currently, insulin administration in research animals is done to comply with guidelines set by animal welfare authorities and usually depends upon the habits and means of the different laboratories. It is thought so unimportant that it is scarcely described in publications [14]. However, from a previous work, we highlighted that global and organ-specific stress and inflammation are dependent upon glycaemia. Indeed, when glycemia was regulated, a decrease in oxidative stress and inflammation was observed [15]. In that case, the portal route allows the first insulin clearance by the liver and better regulation. With regard to the administration mode, since continuous insulin delivery permits better glycaemic regulation than injections, the use of pumps in rodent models could have an important impact on inflammation and oxidative stress, especially in the liver and omentum (fat tissue), the principal organs of glycogen storage [15, 16].

In order to identify the impact of the chosen modes of insulin administration, a single injection of long-acting insulin was compared with continuous release of rapid-acting insulin. Either insulin with retarded activity or osmotic pumps provide a continuous insulin diffusion replacing the basal insulin delivered by the pancreas. The impact of these two treatments on rat metabolism was studied, specifically on liver and omental inflammation and oxidative stress.

## 2. Material and Methods

### 2.1. Animals

Males Lewis rats were supplied by Janvier Laboratory (Le Genes St. Isle, France). The rats were housed in standard collective cages under pathogen-free conditions in a temperature-controlled room (23 ± 1°C) with a 12 h light : 12 h darkness cycle. They were fed SAFE-A04 rodent chow (Villemoisson-sur-Orge, France); the food and water were available ad libitum. All experiments were performed according to the National Institutes of Health guidelines (Authorization Number AL/60/67/02/13).

### 2.2. Diabetes Induction

Experimental type 1 diabetes was induced pharmacologically in Lewis rats by a single intraperitoneal injection of streptozotocin (STZ; 75 mg/kg diluted in citrate buffer, pH 4.5; Sigma, St. Louis, MO, USA). Diabetic rats with nonfasting blood glucose values >400 mg/dL, as measured by a blood glucose monitor (AccuCheck, Roche, Basel, Switzerland) and confirmed by a glucose assay kit (RTU® Glucose; bioMérieux, Marcy-l'Étoile, France), were used in the study. The diabetic state of each rat was verified from its C-peptide level. Blood analysis showed a statistically significant reduction of C-peptide levels in STZ-injected rats compared with their control nondiabetic (CTL ND) counterparts (*p* < 0.01). The low levels of C-peptide remained stable throughout the study (day 14 levels: CTL ND: 2500.63 ± 120.54 pmol/L; diabetic: 15.71 ± 7.50 pmol/L; injection: 13.24 ± 5.06 pmol/L; pump: 6.25 ± 4.31 pmol/L).

### 2.3. Pump Preparation

Osmotic pumps (Alzet®, Cupertino, CA, USA) were loaded with Insuman® 400 IU/mL (Sanofi-Adventis, Paris, France) and placed in warm (37°C) saline solution for 24 h prior to implantation in order to be activated. The dose loaded into the pump allowed for a 30-day release of 4 IU/day, according to the technical description furnished by the supplier. Diluted Insuman (120 IU in buffer solution used for insulin pumps (Sanofi-Aventis Deutschland GmbH, Frankfurt am Main, Germany)) was loaded into the pumps, which were set at a pumping rate of 2.5 ± 0.5 *μ*L/h to allow delivery of 3.60 ± 0.72 IU/day of insulin.

### 2.4. Insulin Therapy

Rats were divided randomly into the following four groups: (i) nondiabetic control rats, untreated (CTL ND group, *n* = 6), (ii) diabetic rats, untreated (diabetic group, *n* = 6), (iii) diabetic rats, treated via injections (injection group, *n* = 11), and (iv) diabetic rats, treated via pumps (pump group, *n* = 9). Rats from the Injection group received 4 IU/200 g of body weight per day of a long-acting insulin (100 UI/mL Insulin Lantus; Sanofi-Aventis, France) via a daily subcutaneous (SC) injection. The pump group receive 4 IU/200 g of body weight per day of a short-acting insulin (Insuman) delivered continuously via an osmotic pump (Alzet) placed in the dorsal SC space. Briefly, rats were anaesthetised with isoflurane (Abbott Laboratories, Berkshire, UK) and placed in the prone position. The skin was shaved and an incision was made longitudinally with a scalpel. The preactivated pump was then introduced through the skin incision with its head in the opposite site of the incision. The incision was then stitched up and the rats were placed under a lamp until they awoken. Thereafter, the rats were treated with an antibiotic (5%, 10 mg/kg Baytril®; Bayer, Lyon, France) once daily for 7 days after surgery.

### 2.5. Study Scheme

**Figure 1:**
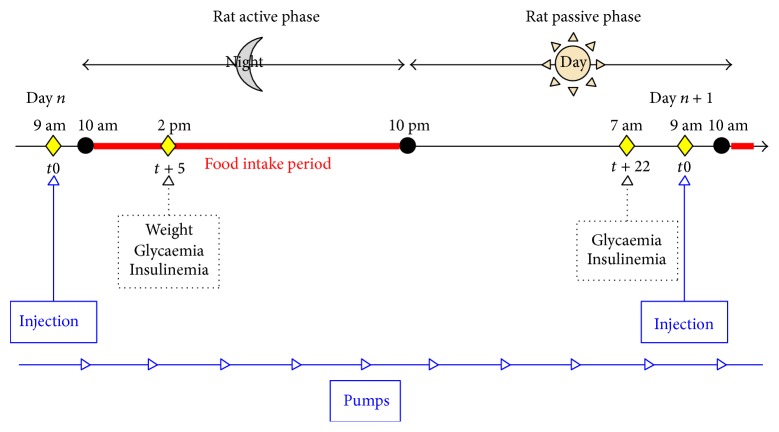
Experimental daily scheme. Blue represents the two insulin administration modes. For the single injection group, 4 UI/200 g of body weight of long-acting insulin was administered each day at 9 am. Analyses were carried out at *t* + 5 and *t* + 22 h thereafter. For the continuous insulin administration group (via pumps), a rapid-acting insulin was loaded into the osmotic pump to allow a delivery of 4 UI/200 g of body weight per day. The injection point *t*0 corresponds to 9 am and analyses were carried out at *t* + 5 and *t* + 22 h thereafter.

Diabetes was induced 1 week before the first injection or pump implantation. Metabolic follow-up was carried out by analysing tail-vein blood, sampled at days 0, 7, 14, 21, and 28 after treatment. The body weight and noninvasive glucose monitoring (AccuCheck) were assessed three times a week. Two time scales were used in this study for assessing the insulinaemia and glycaemia of rats: a short time after SC insulin treatment (namely, 5 h after (*t* + 5 h)) and a long time after treatment (namely, 22 h (*t* + 22 h)). The intraperitoneal glucose tolerance test (IpGTT) was conducted on day 14. The rats were sacrificed on day 28. Total blood was collected into heparinised tubes and then plasma-frozen. Liver and omental tissues were snap-frozen in optimal cutting temperature compound (Tissue OCT, Labonord, Templemars, France) (Figure 1).

### 2.6. Intraperitoneal Glucose Tolerance Test

Intraperitoneal glucose tolerance test (IpGTT) was realized on day 14. Briefly, nonfasting rats were placed in clean cages with no food or feces in hopper or bottom of cage with water access* ab libitum*. Glycaemia was recorded with fingerpick at the end of the tail and blood glucose meter. Baseline glycaemia was measured at *t*0 and then intraperitoneal injection of 2 g of glucose/kg body was realized. Blood glucose levels were measured at 15, 30, 60, and 120 minutes after glucose injection. Food was then reintroduced at the end of the test.

### 2.7. Analysis of Blood Parameters

The blood glucose was assessed using a glucose assay kit (RTU, bioMérieux) and results were expressed in g/L. Rat C-peptide and human insulin were analysed by ELISA (enzyme linked immunosorbent assay, Mercodia, Uppsala, Sweden) and the results were expressed in pmol/L and mU/L, respectively. Insulinaemia and glycaemia status were assessed at *t* + 5 and *t* + 22 h after insulin injection. Plasma fructosamine (expressed in *μ*mol/L) was quantified at the time of sacrifice, using a colorimetric method of Laboratoire Cerba (Cergy Pontoise, France).

### 2.8. Hepatic and Omental Oxidative Stress

The oxidative fluorescent dye dihydroethidine (DHE) was used to evaluate* in situ* formation of reactive oxygen species (ROS), following the method described by Dal-Ros et al. [17]. Unfixed liver or omental tissues were cut into 10 *μ*m thick sections, treated with DHE (2.5 *μ*M), and incubated in a light-protected humidified chamber at 37°C for 30 min. The level of ROS was determined using a microscope and whole fluorescence of tissue was quantified with the microscope assistant (NIS-Elements BR, Nikon, Paris, France). The fluorescence intensity of liver was quantified in five arbitrarily selected fields and the mean value for each section was calculated.

### 2.9. Hepatic and Omental Inflammation

Hepatic and omental macrophage staining was carried out using 10 *μ*m thick tissue sections on slides. In brief, the slides were fixed with 4% paraformaldehyde for 10 min and then incubated with 3% H_2_O_2_ in methanol in order to block endogenous peroxidases. The slides were then blocked and incubated with a 1 : 1000 dilution of rabbit anti-Iba-1 antibody (Wako Chemicals GmbH, Neuss, Germany) for 1 h at room temperature. The biotinylated secondary antibody and reagent solution were provided in the Vectastain Elite ABC kit (Vector Laboratories, Burlingame, CA, USA). Detection was done using the 3,3-diaminobenzidine (DAB) peroxidase substrate kit (Vector Laboratories), and the slides were counterstained with Harris hematoxylin (Labonord, Templemars, France). Observations were made and pictures were taken with a camera-attached microscope (Nikon, France) and macrophage quantification was determined using ImageJ software.

### 2.10. Statistical Analyses

Statistical analyses were performed using Statistica software (StatSoft, Maisons-Alfort, France). Results were analysed by repeated-measures analysis of variance (ANOVA) or one-way ANOVA with a Fisher's least-square difference* post hoc* test. Results are presented as mean ± SEM. A *p* value of less than 0.05 was considered statistically significant. For graphic representation, a *p* value < 0.05 was notified as *∗*, *p* < 0.03 as *∗∗*, and *p* < 0.01 as *∗∗∗*.

## 3. Results

### 3.1. Human Insulin

#### 3.1.1. Short-Time Study

**Figure 2:**
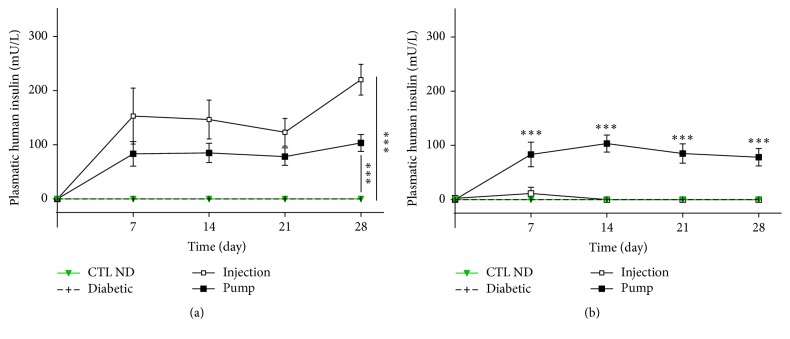
Plasma levels of human insulin at 5 h (a) and 22 h (b) after insulin administration. For both control nondiabetic (CTL ND) and diabetic rats which received no insulin treatment, human insulin was undetectable at *t* + 5 or *t* + 22 h. At *t* + 5 h, both injection and pump rats presented a high level of detectable insulin, but at 22 h thereafter, only the pump group levels continued to increase while the injection group levels did not. CTL ND and diabetic groups are different from injection and pump groups at all times since 7 days (*p* < 0.01) (a); pump group is different from all other groups at all times since 7 days (*p* < 0.01) (b). ^*∗∗∗*^ *p* < 0.01.

At 5 h after injection, human insulin was detectable in both groups of treated rats and was statistically different from the CTL ND rats (day 21 levels: CTL ND: undetectable; diabetic: undetectable; injection: 123.11 ± 25.57 mU/L; pump: 78.19 ± 16.07 mU/L; *p* < 0.01 CTL ND versus injection and pump) (Figure 2(a)).

#### 3.1.2. Long-Time Study

At 22 h after injection, human insulin was detectable only in the pump group and was statistically different from the CTL ND and injection groups (day 21 levels: CTL ND: undetectable; diabetic: undetectable; injection: undetectable except for day 7: 11.27 ± 3.43 mU/mL; pump: 84.99 ± 17.77 mU/L, *p* < 0.01) (Figure 2(b)).

### 3.2. Blood Glucose Levels

#### 3.2.1. Short-Time Study

**Figure 3:**
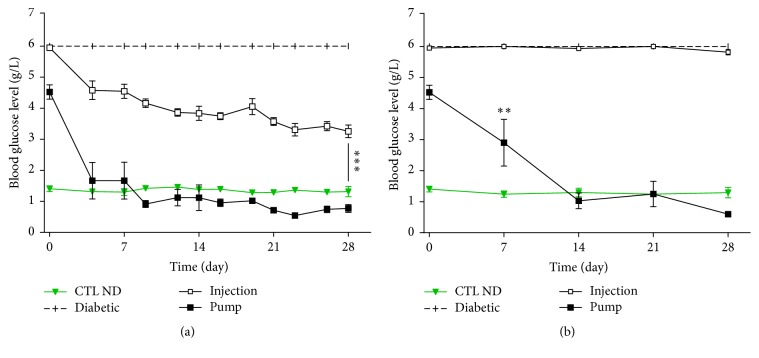
Blood glucose levels at *t* + 5 h (a) and *t* + 22 h (b) after insulin injection. At *t* + 5 h, untreated rats (CTL ND and diabetic) had, respectively, normal glycaemia and 6 g/L glucose, the highest value detectable with our material. Pump rats succeeded in achieving normoglycaemia in a few days, whereas the injection group only reached ~4 g/L. Diabetic group is different (*p* < 0.01) from all groups at all times. Injection group is different (*p* < 0.01) from all other groups at all times since day 4 (*p* < 0.01). Pump group is different (*p* < 0.01) from injection and diabetic group since day 4 (a). On a longer time (*t* + 22 h), CTL ND rats still had a normal blood glucose level, whereas the diabetic and injection groups levelled at 6 g/L. Pump rats still achieved normoglycaemia. Diabetic and injection groups are different (*p* < 0.01) from all groups at all times. ^*∗∗*^ *p* < 0.03 pump versus CTL ND at day 7 (b). ^*∗∗∗*^ *p* < 0.01.

The blood glucose levels were controlled by insulin (whether continuously delivered or by injection, Figure 3(a)) at 5 h after injection as compared with that at *t*0. Rats in the pump group were normoglycaemic (below 1.20 g/L) throughout the study, whereas the glucose levels of rats in the injection group increased dramatically to ~4 g/L (day 21 levels: CTL ND: 1.28 ± 0.02 g/L; diabetic: 6.00 ± 0.00 g/L; injection: 4.05 ± 0.26 g/L; pump: 1.02 ± 0.07 g/L) (Figure 3(a)).

#### 3.2.2. Long-Time Study

After 22 h, rats in the pump group were still normoglycaemic, whereas the blood glucose levels of rats under insulin injection therapy were comparable to those of untreated diabetic rats (day 21: CTL ND: 1.24 ± 0.02 g/L; diabetic: 6.00 ± 0.00 g/L; injection: 6.00 ± 0.02 g/L; pump: 1.25 ± 0.41 g/L) (Figure 3(b)).

### 3.3. Weight

**Figure 4:**
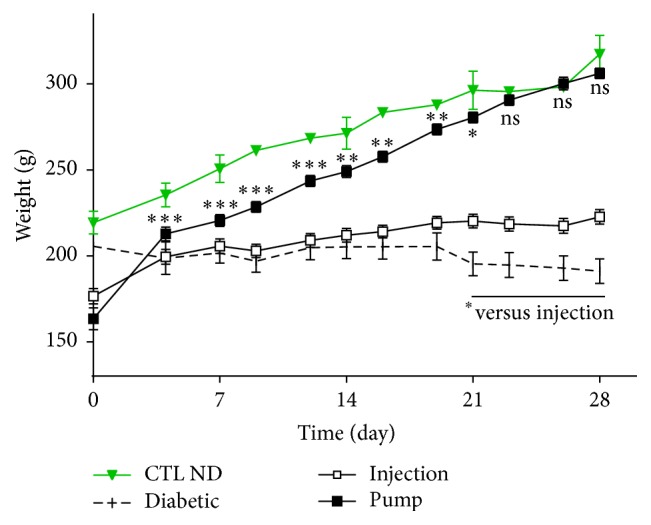
Weight of rats. The control nondiabetic (CTL ND) rats had a normal and regular weight gain curve. Rats of the pump group succeeded in reaching the control level within 21 days. Rats from the diabetic and injection groups did not gain much weight during the study period. Injection group is different from CTL ND and pump groups at all time points since day 7 (*p* < 0.01). Injection group is also different from diabetic group since day 21 (*p* < 0.05). Pumps versus CTL ND: ns = non significant. ^*∗*^ *p* < 0.05, ^*∗∗*^ *p* < 0.03, and ^*∗∗∗*^ *p* < 0.01.

The weight of rats on insulin therapy increased throughout the study, whereas that of untreated diabetic rats remained relatively unchanged. Weight gain was more prominent in the pump group (reaching the level of the CTL ND group) than in the injection group. During the first 8 days, the weight of rats in the pump group was significantly lower than that of CTL ND rats (*p* < 0.01). Over the next few days (12–19 days), the pump group weight levels were still lower than the controls but to a lesser degree (*p* < 0.05), and by the end of the study (21–28 days) there was no statistically significant difference between these two groups (Figure 4). The weight measure in the injection group was different from the controls throughout the follow-up period (0–28 days, *p* < 0.01) and was significantly lower than the pump group from day 4 onwards (4–28 days, *p* < 0.01).

### 3.4. Intraperitoneal Glucose Tolerance Test

**Figure 5:**
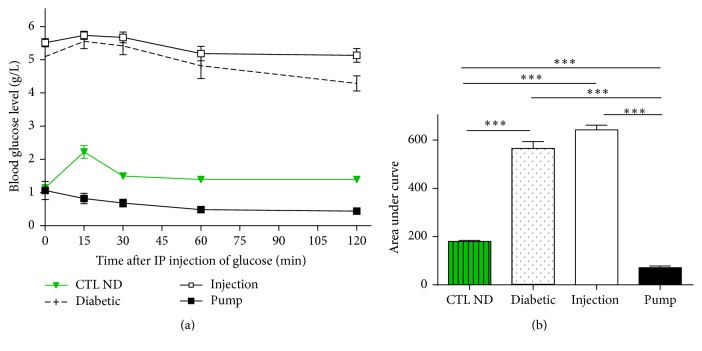
Intraperitoneal glucose tolerance test on rats. IpGTT profile (a) and areas under the curves (b). In control nondiabetic (CTL ND) rats, a rapid elevation of blood glucose (within 15 min) was observed, but with a rapid return to basal level. Diabetic and injection rats had similar profiles, with the glucose levels being much higher than the basal level and remaining high for more than 30 min. For the pump rats, glycaemia was observed only after glucose infusion. IP glucose, intraperitoneal glucose injection; IpGTT, intraperitoneal glucose tolerance test. ^*∗∗∗*^ *p* < 0.01.

Both the injection and pump groups did not have a normal IpGTT response profile. The CTL ND group responded properly, with an initial increase of glucose level at 15 min (2.22 ± 0.19 g/L), followed by a progressive decrease at 30 min (1.50 ± 0.08 g/L), and finally reaching the normal level by 60 min (1.39 ± 0.05 g/L). The diabetic group was unable to respond properly (*t*0 min: 5.09 ± 0.04 g/L;* t*15 min: 5.55 ± 0.22 g/L;* t*60 min: 4.82 ± 0.23 g/L). The injection group did not respond to glucose (*t*0 min: 5.51 ± 0.12 g/L;* t*15 min: 5.74 ± 0.12 g/L) and was also not able to reduce the levels after glucose injection (*t*60 min: 5.13 ± 0.21 g/L). In the pump group, no glucose elevation was observed (*t*0 min: 1.06 ± 0.28 g/L;* t*15 min: 0.82 ± 0.16 g/L), and the levels remained stable during the 2 h of experiment (~1 g/L) (Figure 5).

### 3.5. Fructosamine Levels

**Figure 6:**
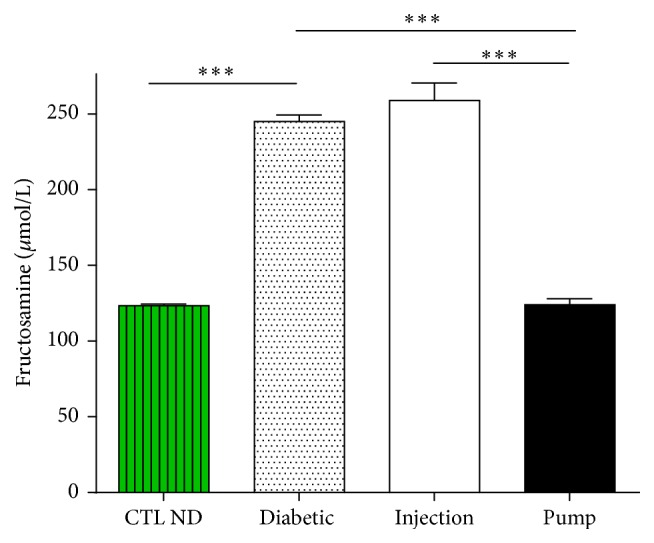
Plasma fructosamine level at time of sacrifice (30 days). Diabetic rats had a higher level of fructosamine than control nondiabetic (CTL ND) rats. Injection rats had a level comparable to the diabetic rats, whereas the level in pump rats was the same as CTL ND rats. ^*∗∗∗*^ *p* < 0.01.

Fructosamine levels reflect the glycaemic balance in rats. The levels in the pump group were comparable to those of the CTL ND group (CTL ND: 126.00 ± 5.37 *μ*mol/L; pump: 124.00 ± 4.03 *μ*mol/L, *p* > 0.05). The levels in the Injection group were statistically higher than in the CTL ND or pump groups (injection: 258.88 ± 11.52 *μ*mol/L, *p* < 0.01) but no difference was observed between the diabetic and injection groups (diabetic: 245 ± 4.29 *μ*mol/L, *p* > 0.05) (Figure 6).

### 3.6. Liver and Omental Oxidative Stress

**Figure 7:**
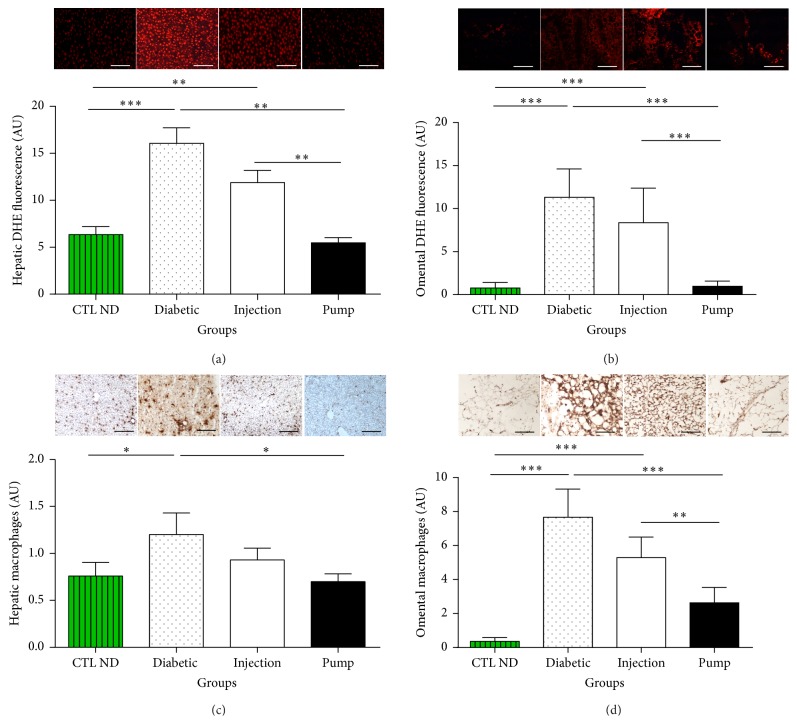
Reactive oxygen species (ROS) staining in liver (a) and omental tissue (b), macrophage staining in liver (c) and omental tissue (d), and their respective quantifications. The profiles were similar for all quantitative graphs, with diabetic rats having the highest level of oxidative stress or inflammation in both tissue sites. The control nondiabetic (CTL ND) and pump rats had the lowest level of ROS and macrophage staining. The injection group had lower levels than the diabetic group, but higher levels than the CTL ND or pump groups. ^*∗*^ *p* < 0.05, ^*∗∗*^ *p* < 0.03, and ^*∗∗∗*^ *p* < 0.01. Scale bar: 100 *μ*m. DHE, dihydroethidine.

The liver and omentum were chosen to study oxidative stress because they represent glucose storage organs and can be directly affected by chronic hyperglycaemia. In addition, the tissues represent the actual and an alternative site for islet transplantation, respectively. Basal fluorescence intensity was observed in the liver tissue of CTL ND rats (6.35 ± 0.85 AU). Compared with the controls, the level was significantly higher in the diabetic group (16.06 ± 1.67 AU, *p* < 0.01) and moderately higher in the injection group (11.88 ± 1.31 AU, *p* > 0.05). That of the pump group was comparable to the CTL ND group (5.48 ± 0.55 AU, not significant (ns)) (Figure 7(a)).

Similar patterns were observed in the omentum: that is, a basal level of fluorescence intensity in the CTL ND rats (0.78 ± 0.44 AU), a significantly increased level in the diabetic group (11.31 ± 5.657 AU, *p* < 0.01), a moderately higher level in the injection group (8.35 ± 4.01 AU, *p* > 0.05), and a comparable level in the pump group relative to the control rats (0.96 ± 0.36 AU, ns) (Figure 7(b)).

### 3.7. Liver and Omental Inflammation

The level of macrophages in the liver and omentum reflects the inflammation status in these tissues.

Macrophage staining was observed in the liver of CTL ND rats (0.76 ± 0.27 AU), and this level was comparatively much higher in the diabetic group (1.20 ± 0.23 AU, *p* < 0.05), moderately higher in the injection group (0.93 ± 0.13 AU, *p* > 0.05), and of comparable level in the pump group (0.7 ± 0.08 AU, ns) (Figure 7(c)).

In the omentum, a similar pattern of macrophage staining was observed (CTL ND: 0.35 ± 0.22 AU; diabetic: 7.66 ± 1.65 AU, *p* < 0.01; injection: 5.29 ± 1.20 AU, *p* > 0.05; and pump: 2.62 ± 0.90 AU, ns) (Figure 7(d)).

## 4. Discussion

In this study, we showed that the continuous release of insulin with pumps controlled glycaemic fluctuations, which improved the metabolic parameters of oxidative stress and macrophage infiltration in both the liver and omentum. On the contrary, insulin injections did not ensure glycaemic regulation and showed a short efficiency time of the insulin despite its long-acting characteristic. Subsequently, variations in the blood glucose levels enhanced the oxidative stress and inflammation status of the liver and omentum.

We chose the same dose for both the once-a-day long-acting injected insulin and the continuously administrated rapid-acting insulin in the hope that the effects would be similar. In fact, long-acting insulin (or better named extended-release insulin) has a delay of action of 1 h and duration of 24 h. This kind of insulin is obtained through the modification of amino acids. It is able to precipitate in the SC space through modification of the pH to 6.7 and is less soluble at physiological pH, which explains its slow action rate. Normally, this insulin can achieve a peak level for at least 24 h and is nondetectable after 22 h.

Nevertheless, the continuous delivery of small amounts of rapid-acting insulin, also designed for human use, achieved better glycaemic regulation throughout the day. The one used in our study is Insuman, which is unmodified insulin with rapid action (10–15 min) and short duration (5–8 h) times. Theoretically, both insulin types should have decreased the blood glucose levels throughout the day. The difference observed between Lantus and Insuman could perhaps have resulted from the chemical modification. In fact, modified insulin will not bind to insulin receptors with the same affinity as unmodified insulin [18]. Furthermore, the insulin used is adapted to humans, not to rats, so the structural difference and lower affinity for insulin receptors could be explanations for the lower effective action recorded. To achieve glycaemic control by injection, the doses or the number of injections should be increased [19].

The improvement of glycaemic fluctuations by a continuous release of insulin was probably due to several factors. The constant amounts of rapid-acting insulin released guaranteed the continuous presence of insulin in the blood. This has been described to be more efficient than injections in humans [9] and, as shown in our model, likewise in rats. In the present work, injections were less efficient. The dose of insulin delivered could have been variable since it was dependent upon the manipulation, and the repetitive injections could have triggered fibrosis which prevents proper diffusion of the insulin [20]. As a result, we found that the weight gain was lower in the injection group. However, continuous insulin diffusion needs to be optimised since, in our model, the regulation of glycaemia was too efficient and the blood glucose level was very low. This large amount of insulin release could create chronic hyperinsulinism. This was highlighted especially by the absence of glucose increase in the IpGTT test. Hyperinsulinism could also have side effects on rat metabolism [21], such as triggering insulin resistance [22–24]. In our study, however, no insulin resistance was detected as glycaemia and insulinaemia were stable throughout the study. Nevertheless, to avoid insulin resistance and to be able to control glycaemia, a 3 IU/day dose seems to be the best option for a continuous SC administration, since 2 IU had been invalidated in a previous study [15].

The decrease in glycaemic fluctuations decreased oxidative stress (presence of ROS) and inflammation (presence of macrophages) in the liver and omentum. This was in accordance with previous results stating that a better regulation of the blood glucose level using the continuous administration mode had a positive effect on oxidative stress and inflammation in the liver [15]. By comparing the difference in glycaemic fluctuations between the pump and injection groups, we were able to identify a cause of oxidative stress and inflammation in the tissues. Glucose is known to be prohypertensive [25] and proinflammatory [26, 27], as proved by the increase in liver and omental macrophage infiltrations in this study. Furthermore, cells which are incubated with a high concentration of glucose tend to secrete more cytokines for a same stimulus, suggesting an underlying indirect mechanism for the glucose-induced inflammatory reaction [28]. Indeed, injections result in a variation in glucose levels, as shown by the much higher fructosamine levels, equivalent to Hb1Ac, which was a generator of oxidative stress. On the contrary, the stability of glycaemia in the pump group resulted in a significant decrease in fructosamine levels and significant decrease in oxidative stress.

The liver and omentum were chosen because they are the primary glucose storage sites and because they are the classical and a promising alternative site for islet transplantation, respectively [29]. The characterisation of these two sites has never been done in terms of inflammation and oxidative stress in relation to glycaemic regulation. Indeed, in this study, we showed that high glucose fluctuations increased oxidative stress in both the liver and omentum. During islet transplantation, it has been shown that the graft can be highly sensitive to inflammation and oxidative stress [30–32] which could lead to an early loss of the transplanted tissue. This study highlights the importance of glycaemic regulation before islet transplantation, since it can be critical for engraftment outcome. Oxidative stress is particularly relevant and dangerous for the transplanted islets, which are among those tissues that have the lowest levels of intrinsic antioxidant defenses [33]. As shown in the present study, continuous delivery of insulin was able to maintain normoglycaemia and consequently decreased oxidative stress and inflammation. As we showed, these two sites were rich in ROS in the diabetic state, and although endogenous ROS in physiological concentrations do help to maintain homeostasis, they can still cause chronic oxidative stress and adverse effects when they accumulate. It is well described that diabetes complications are generated by a high level of circulating glucose. Some studies [34] have highlighted the loss of animals as a result of severe hyperglycaemia complications and the dilemma of having to keep animals in a diabetic state for a long period while waiting for islets from, for example, human material. At this time, pumps seem to be the best option, because they can be removed at any time and can keep animals in good health while waiting for available material for islet transplantation, by limiting the adverse effects of diabetes. This study highlights the need to properly manage the diabetes before transplantation in order to allow islets to be engrafted in a healthy environment without glucotoxicity, oxidative stress, and inflammation [35].

In addition, this study was important for highlighting the differences observed in terms of metabolism, inflammation, and oxidative stress in rats treated with the same amount of insulin, a comparison that would not have been possible using different insulin doses. In fact, in diabetes studies, researchers use many different ways to implement insulin therapies. Firstly, the type and the origin of insulin types vary: slow or rapid, human, porcine, and so forth. Secondly, doses are also not so well defined, being anywhere from 1 IU to 15 IU [36]. Making the comparison even more complex, several models of pumps exist (osmotic pumps [37, 38] or preloaded implants, also named insulin pellets [35, 39]). Unlike the case of preloaded insulin pellets, osmotic pumps need to be filled manually. Although these main differences may seem to be minor, they are in fact highly important in comparison studies, as proven by the differences observed in this study under treatment conditions that appear to be the same.

In conclusion, our comparison of the two modes of insulin delivery promotes the use of continuous insulin administration, in terms of feasibility and good results. In fact, pumps require no daily injection and facilitate rat follow-up. The well-being of the animals and the homogeneity of the results permit researchers to limit the numbers of animals and experiments used to build solid and reproducible results, as confirmed by other studies [40]. Continuous insulin release has a positive effect on rat metabolism (glycaemia, weight gain) and can limit adverse effects of the diabetic state (oxidative stress and inflammation). Additionally, this study highlights the limitations of comparative studies, as different insulin therapies could modify the metabolic parameters.
